# Epicardial Versus Endocardial Pacing in Paediatric Patients with Atrioventricular Block or Sinus Node Dysfunction: A Systematic Review and Meta-analysis

**DOI:** 10.1007/s00246-023-03213-x

**Published:** 2023-07-22

**Authors:** Vasiliki Patsiou, Anna-Bettina Haidich, Amalia Baroutidou, Andreas Giannopoulos, George Giannakoulas

**Affiliations:** 1 First Department of Cardiology, AHEPA University Hospital, School of Medicine, Faculty of Health Sciences, Aristotle University of Thessaloniki, Thessaloniki, Greece; 2 Department of Hygiene, Social-Preventive Medicine and Medical Statistics, School of Medicine, Faculty of Health Sciences, Aristotle University of Thessaloniki, 54124 Thessaloniki, Greece; 3 Second Department of Pediatrics, AHEPA University Hospital, School of Medicine, Faculty of Health Sciences, Aristotle University of Thessaloniki, Thessaloniki, Greece

**Keywords:** Pacemaker, Epicardial, Endocardial, Atrioventricular block, Sinus node dysfunction, Children, Pediatric

## Abstract

Pacing indications in children are clearly defined, but whether an epicardial (EPI) or an endocardial (ENDO) pacemaker performs better remains to be elucidated. This systematic review and meta-analysis aimed to directly compare the incidence of pacemaker (PM) lead-related complications, mortality, hemothorax and venous occlusion between EPI and ENDO in children with atrioventricular block (AVB) or sinus node dysfunction (SND). Literature search was conducted in MEDLINE (via PubMed), Scopus by ELSEVIER, Cochrane Central Register of Controlled Trials (CENTRAL), Web of Science, and OpenGrey databases until June 25, 2022. Random-effects meta-analyses were performed to assess the pacing method’s effect on lead failure, threshold rise, post-implantation infection and battery depletion and secondarily on all-cause mortality, hemothorax and venous occlusion. Several sensitivity analyses were also performed. Of 22 studies initially retrieved, 18 were deemed eligible for systematic review and 15 for meta-analysis. Of 1348 pediatric patients that underwent EPI or ENDO implantation, 542 (40.2%) had a diagnosis of congenital heart disease (CHD). EPI was significantly associated with higher possibility of PM-lead failure [pooled odds ratio (pOR) 3.00, 95% confidence interval (CI) 2.05–4.39; *I*^2^ = 0%]; while possibility for threshold rise, post-implantation infection and battery depletion did not differ between the PM types. Regarding the secondary outcome, the mortality rates between EPI and ENDO did not differ. In sensitivity analyses the results were consistent results between the two PM types. The findings suggest that EPI may be associated with increased PM-lead failure compared to ENDO while threshold rise, infection, battery depletion and mortality rates did not differ.

**Supplementary Information:**

The online version contains supplementary material available at 10.1007/s00246-023-03213-x.

## Introduction

Bradycardia caused by high-degree atrioventricular block (AVB) or less often sinus node dysfunction (SND) is the most prevalent indication for permanent cardiac pacing in pediatric patients [1]. To date, two established approaches of cardiac pacing exist: the epicardial (EPI) and endocardial (transvenous) (ENDO) route. However, which pacing method should be preferrable in children remains controversial. Of note, several challenging pediatric issues, such as patient somatic growth, anatomy, concomitant CHD with or without intracardiac shunt and presence of vascular access to the heart, must be taken into account when deciding the optimal pacing method [2–4]. Due to the potential for lifelong stimulation, higher pacing standards are required, particularly for children of younger ages.

Moreover, pediatric patients represent a small subpopulation, accounting for less than 1% of all patients who undergo permanent pacemaker (PM) implantation [5]. Thus, despite the growing experience of centers and improvements in pacing devices the last decades, no customized hardware has been designed for children and the number of PM revisions is still considerable [6]. Data from studies that compared the adverse events of EPI and ENDO in children are contradictory. Both pacing methods have presented device-related complications, such as lead and battery failure [7], that often lead to reoperations, and some severe but rather rare adverse events such as venous occlusion [8], hemothorax [9] and cardiac strangulation [10] that can lead to coronary compression [11]. Evidence on device- related mortality rates is little and non-conclusive. Nevertheless, the therapeutic benefit of the PMs in pediatric AVB or SND could not be undermined especially after the establishment of steroid-eluting leads that have upgraded the performance of both PM types [12]. Yet, criteria such as weight, age and body surface that are usually chosen [13, 14] to classify children in the optimal pacing method remain largely dependent on expert recommendations and centers’ experience.

This systematic review and meta-analysis aimed to summarise the available evidence regarding the performance of EPI and ENDO PMs in children and their potential association with adverse events. The comparison of single or dual-chamber pacing was not discussed in this study.

## Methods

The current systematic review and meta-analysis was performed in accordance with the Preferred Reporting Items for Systematic reviews and Meta-Analyses (PRISMA) guidelines [15]. The pre-specified research protocol has been registered a priori in the PROSPERO database (CRD42022366894: https://www.crd.york.ac.uk/prospero/display_record.php?RecordID=366894).

### Literature Search

The following electronic databases were independently searched by three reviewers (VP, ABH, AB) from inception until June 2022: PubMed, Scopus, Cochrane Central Register of Controlled Trials (CENTRAL), Web of Science and OpenGrey. Τhe search strings included the following Basic keywords used in search strings were “epicardial”, “endocardial”, “pacemaker”, “sinus node dysfunction”, “atrioventricular block” and “pediatric” and both free text and Medical Subject Headings (MeSH) format was used. Finally, hand-searches were conducted on the reference lists of the eligible studies and relevant reviews to identify additional papers.

### Studies Eligibility Criteria

In this meta-analysis, observational (prospective or retrospective) cohort or randomized controlled studies were included if they compared the risk of clinical outcomes between pediatric individuals with AVB or SND that underwent EPI implantation with those who underwent ENDO implantation. Specifically, studies were considered eligible if > 75% of the participants were children with AVB or SND and PM implantation, and > 90% of them, were < 18 years old.

The following studies were excluded from the meta-analysis: (a) abstracts, case reports, reviews, editorials, and practice guidelines; (b) studies that did not specifically report event rates, hazards or risk ratios, or 95% confidence intervals (CIs) for outcome measurements; (c) studies involving only one PM type and (d) articles published in languages other than English were also excluded.

### Data Extraction and Quality Evaluation

Data from the eligible studies were reviewed and extracted independently by two authors. An electronic data extraction sheet from Excel was used to collect and manage the following information: study design, sample size, population characteristics, follow-up period, concomitant congenital heart defects, and causes of PM revisions/reoperations. Missing data were handled by contacting the corresponding study authors via email to obtain any significant information.

The methodological assessment of the included studies was performed for the primary outcomes using the ROBINS-I tool, which is appropriate for non-randomised studies, according to the Cochrane Handbook for the Systematic Reviews of Interventions version 6.3.0 [16]. Two independent reviewers conducted the data extraction and quality assessment (VP and AB). Any disagreements were adjudicated by a third reviewer (GG). The evaluation of the quality of evidence was conducted using the “Grading of Recommendations, Assessment, Development and Evaluations (GRADE)” [17] approach. Possible publication bias was assessed using funnel plots asymmetry and was tested with the Harbord’s test [18] using R version 4.2.1 for outcomes reported in at least ten studies. Due to the small number of the studies included in each analysis, funnel plots were avoided, and the Harbord’s test was applied only in the outcome of PM lead failure.

### Outcomes

#### Primary Outcomes

The primary study outcomes were PM-lead failure (defined by insulation break, fibrosis, exit block, fracture or other unspecified causes that led to lead malfunction), threshold rise, infection, and battery depletion incidence which was defined as the premature need for battery replacement.

#### Secondary Outcomes

The secondary outcomes included mortality rate, hemothorax and venous occlusion.

### Atrioventricular Block and Sinus Node Dysfunction Definition

The term “atrioventricular block” in this study refers to any degree of congenital or acquired heart block (1st, 2nd, or 3rd) resulting in interruption or delay of electrical conduction in children, while the term “sinus node dysfunction” refers to cases with the following findings on the electrocardiogram: “(1) sinus arrest for more than 2 s, (2) sinus bradycardia (less than 50 beats/min), (3) the tachycardia-bradycardia syndrome and (4) sinoatrial block.”

### Data Synthesis and Analysis

Rates of events have been recorded for both intervention (EPI children with AVB or SND) and control (ENDO children with AVB or SND) groups. Random effects meta-analysis was chosen as high clinical and statistical heterogeneity was expected among the selected studies because of the different therapeutic protocols followed in the included pediatric populations and the different follow-up periods. The counting unit that was used for the pooled results was the “PM system” as it was reported by the majority of studies. All analyses were performed using Review Manager (RevMan) [Computer program]. Version 5.4, The Cochrane Collaboration, 2020. Statistics for binary outcomes are expressed as the pooled odds ratio (pOR). The effect sizes for both types of data are expressed with 95% confidence intervals (CI). Tests of heterogeneity were conducted by calculating *I*^2^, with *I*^2^ > 50% indicating significant heterogeneity.

### Sensitivity Analyses

As the expected heterogeneity of the included studies was considerable, sensitivity analyses were performed to correlate the outcomes with the individual study design characteristics. In particular, the impact of studies with a substantial proportion (> 30%) of SND patients as well as those without clearly PM-related adverse events was investigated.

## Results

### Study Selection and Study Characteristics

A total of 62 articles was initially retrieved. Ultimately, 18 studies satisfied the predefined eligibility criteria and were included the systematic review, while 15 were included in the meta-analysis. The detailed flow diagram of the study selection process is presented in Figure S1. Among the included studies, no randomized clinical trials were found relevant to our topic. All 18 studies that met the inclusion criteria and thus were included in the systematic review were observational and retrospective except of two cross-sectional [19, 20].

A total of 1348 pediatric patients were included in the selected studies. 40.2% of them (542 patients) had a structural heart defect diagnosed before baseline assessment. Of them, ventricular septal defect was the most encountered (~ 36%). The sample sizes, as well as the time of outcome measurements and follow-up periods, widely varied across studies (Table S1).

Of patients, 40% were females. Mean implantation age was 6.8 years. Specifically, for EPI group it was 2.0 ± 4.4 years while for ENDO 5.0 ± 8.1 years. Mean body weight was 25.7 kg (EPI:14 ± 20 kg ENDO:45 ± 15 kg). Table S2 summarizes the main characteristics of the selected patient populations. Congenital AVB was the most common indication for PM implantation (43.2%), followed by acquired ABB which was present in approximately 1 out of three children (34.2%). SND was reported in 243 children (18%) while other indications constituted a smaller proportion (< 10%). No data regarding the pacing method according to each specific dysrhythmia were reported in the pool of studies.

### Risk of Bias Assessment

The risk of bias assessment, which was conducted using the ROBINS-I tool for the included studies, is shown in Figure S2. Included studies showed overall moderate quality.

### Publication Bias

To assess the potential publication bias, Harbord’s test was used for funnel plot asymmetry, Regarding PM lead failure, there was no evidence of publication bias (*P* = 0.56).

### Primary Outcomes

#### Incidence of PM Lead Failure

Thirteen studies (*n* = 1134 patients) reported results regarding the incidence of PM lead failure (including insulation, fibrosis, exit block, fracture, or other unspecified causes). A meta-analysis of the incidence of PM lead failure between EPI and ENDO showed that a PM lead failure in EPI is three-fold compared to ENDO (95% CI of 2.05–4.39, *P* < 0.001), (Figure S3). No statistical heterogeneity was observed among the included studies (*P* < 0.001, *I*^2^ = 0%).

#### Threshold Rise

Five studies (*n* = 206 patients) reported the incidence of threshold rise among the two pacing groups. The comparison of the threshold rise among the two PMs resulted in an OR of 3.12 (95% CI 0.92–10.57, *P* = 0.07, Figure S4), demonstrating a trend of a higher possibility of threshold rise in the EPI group than that in the ENDO group. Statistical heterogeneity was low (*P* = 0.29, *I*^2^ = 19%).

#### Post-implantation Infection

Among seven studies (*n* = 391) reporting the infection after PM implantation. No difference in the possibility of infections between the two PM groups (OR 0.60, 95% CI 0.22–1.62, *P* = 0.31) was found (Figure S5). There was no statistical heterogeneity among the studies (*P* = 0.57, *I*^2^ = 0%).

#### Battery Depletion

Five studies (*n* = 506) reported data on PM replacements due to battery depletion. A random-effects meta-analysis of battery depletion between PMs resulted in a collective OR of 1.47, with a 95% CI of (0.35, 6.12), *P* = 0.60 (Figure S6), indicating no difference between EPI versus ENDO with respect to this outcome. Statistical heterogeneity was considerable (*P* = 0.003, *I*^2^ = 75%).

### Secondary Outcomes

#### Mortality Rates

Six studies (*n* = 568) reported results on mortality rates sufficient for meta-analysis. These studies had a moderate percentage of statistical heterogeneity (*P* = 0.82, *I*^2^ = 59%). A meta-analysis of post-implantation mortality rates between the two groups of PMs resulted in a pooled OR of 1.25, (95% CI of 0.18–8.74, *P* = 0.82) and it is shown in Figure S7. Hence, there is no significant difference in the probability of death between EPI and ENDO.

#### Hemothorax and Venous Occlusion

Two studies (*n* = 381) reported data regarding the incidence of hemothorax. In these studies, 196 children were implanted with an EPI and 185 with an ENDO. Of them, six patients presented hemothorax all, from the group of ENDO. Due to the fact their number is < 3 we did not proceed to a meta-analysis. Udink et al.[21] reported that four children with EPI presented post-pericardiotomy syndrome while another one, pneumothorax. Among the included studies, Wilhelm et al. [7] was the only one which assessed venous occlusion. Eleven children (13.7%) experienced venous occlusion (3 EPI, 8 ENDO) with nine of them presenting no relevant symptoms.

### Sensitivity analyses

Regarding the results of the meta-analyses, it was evident that the comparison of battery depletion outcome between EPI and ENDO demonstrated substantial heterogeneity (75%) indicating variability in the data. Therefore, a sensitivity analysis was conducted, excluding the study by Silvetti et al. [9] a study with a broader age range and a higher mean follow-up duration compared to the others. Moreover, it was the only study with different population, as Silvetti et al. included SND patients in 40% who mostly had an ENDO (> 50%) implanted. On the contrary, all the other included studies had SND patients in a percentage < 10%. The sensitivity analysis included 4 studies and the result was confirmed as no significant difference (*P* = 0.49) was found between EPI and ENDO incidence of battery depletion.

Another sensitivity analysis which was considered reasonable was conducted for mortality rates. The studies of Wilhelm et al. and Beaufort-Krol et al. [22] were excluded as their reported data was not clear regarding PM-related mortality rates. Lotfy et al. was also excluded due to great difference between the number of children to each group. (11 vs 80). The sensitivity analysis included three studies and the results were in line with the initial meta-analysis results. Specifically, no significant difference (*P* = 0.79) was revealed between EPI and ENDO among the three studies that were included in the sensitivity analysis (Figure S7), while heterogeneity was reduced *I*^2^ = 32%. The two sensitivity analyses are shown in figures S8 and S9 respectively.

## Discussion

To date, children with AVB or SND that are needed to be implanted with a PM are treated according to their physical and anatomical characteristics, especially children with CHDs due to their structural and vascular malformations that are often concomitant or the cause of such arrhythmias. The decision involves many challenges and potential adverse events [23, 24]. To our knowledge, this is the first meta-analysis evaluating the PM-related complications and mortality rates in children following EPI or ENDO implantation. Among 1348 pediatric patients with AVB or SND that underwent PM implantation, several interesting findings were noted: (1) EPIs had a threefold increased risk of PM-lead failure compared to ENDOs, (2) the risk of post-implantation infection, threshold rise, battery depletion and mortality did not differ between the two groups.

The present meta-analysis shows a relationship between the EPI pacing method and the occurrence of PM-lead failure while there was no evidence that mortality risk differed between the two pacing methods. These are relevant factors to incorporate into counselling about pediatric PM implantation and suggest that there is room for improvement in epicardial systems. However, previous studies have reported that the use of steroid-eluting leads seems to solve, to a great extent, issues related to sensing and threshold rise, especially in EPIs [25–27]. Particular attention is required for patients with CHDs, as often, due to structural vascular defects (i.e., intracardiac shunts) and possible prosthetic valves, epicardial pacing is the preferred method, preserving transvenous access.

Regarding the primary outcomes, EPIs were found inferior to ENDOs when compared for PM-lead failure. Moreover, although ENDOs were not correlated with an increase in the incidence of infections in our analysis, in previous studies it has been discussed that there might be an association between ENDOs and post-implantation infections [28–30] and indicated that preventive measures should be considered when this route was chosen. It is of interest, that battery depletion, constituted the main reason for reoperations in one-third of the studies. This indicates that further studies are needed to focus on future battery-free devices [31, 32]. Although the comparison regarding reoperations between EPIs and ENDOs was not an outcome of the current study, the individual potential need for reoperations is important to consider in deciding on the most appropriate pacing method, as the reoperation rates remain at high levels and affect the clinical course of children [7, 33, 34].

Existing literature about mortality and PMs among childern is in concordance with our results [1, 35–37]. Several factors such as older age, CVD, heart failure and cardiomyopathies have been highlighted to be correlated with short- or long-term post-implantation mortality [38–41]. Concerning hemothorax events, according to previous literature, their occurrence is rare. However, in line with previous studies [42–44], the two of our included studies that provided data about hemothorax, reported that all the events occurred after ENDO implantation. Also, to date, venous occlusion, which is also a major concern [45] that follows transvenous implantation, is not adequately reported by studies. In our meta-analysis only, Wilhelm et al. described childrens’ cases with venous occlusion concerning mostly the ENDO PMs. In fact, the aim to circumvent such complications, which may be associated with transvenous systems, greatly determines the decision-making process [8, 46–49]. Recent studies report that leadless-pacemaker implantation is largely preferable to be performed in adult patients, and only a small number of successful pediatric cases have been reported until today [50–53].

Regarding the difference between EPI and ENDO in children’s baseline characteristics such as age, weight, and body surface that, according to literature [54], are used as definitive for the decision on pacing method, the data were quite heterogenous for comparison thus, drawing a robust conclusion was not possible. However, younger age has been associated with a higher complication rate during follow-up, regardless of the pacing method [55–58]. According to previous studies [59–61], EPI was preferred for the majority of younger children and children with lower body weight (< 15 kg) with structural vascular lesions, leaving ENDO as a primary choice for older pediatric patients with higher body surfaces and normal cardiovascular structure. Admittedly, in these studies, apart from the anatomical such as rapid growth, generator pocket placement due to their small size, confinement in the access to the atrium or the ventricle (i.e., Fontan patients), and somatic factors there were unmeasured confounders that might have a different impact on pacing method decision among children with AVB or SND. such as the doctor’s experience, parents’ opinion, access to necessary for each technique materials, and even socioeconomic factors. Nevertheless, currently, research seems to primarily focus on the optimal pacing sites in children, which have been previously attempted in adults, regardless of the EPI or ENDO method [62–66].

In conclusion, the PM system and leads, as well as the implantation route, are still determined by somatometric measurements and the anatomy of the child without certain guideline-indicated criteria. The results of this meta-analysis suggest that the EPI pacing method may be associated with higher possibility of PM lead failure and constitutes a question for future research to investigate potential solutions to prevent them. More large-scale and multicenter studies should focus on the optimal pacing method in children in order to significantly reduce reoperations and optimize the PM system hardware increasing its compatibility with children’s needs.

### Limitations

This systematic review and meta-analysis has several limitations. The main limitation of this review is that included only observational studies, which are prone to biases and confounding as no multi-variable adjustments were applied, thus undermining their internal validity. Nevertheless, their external validity increases the generalizability of the systematic review. Regarding our results, should be interpreted in the context of the relatively small sample sizes among the included studies.

Moreover, the comparison between dual and single chamber PMs, although a considerable pacing factor, was not feasible regarding the studies’ provided data.

Another limitation was the heterogeneity between the studies in relation to the reported baseline characteristics. For this reason, a comparison between EPI and ENDO on the mean weight and age of children, which would lead to a conclusion about which PM is chosen based on these characteristics was not feasible. To overcome the heterogeneity across studies regarding the outcomes, we had pre-specified sensitivity analyses to be conducted.

## Conclusions

Permanent pacing therapy in the young remains a puzzling issue. This systematic review and meta-analysis concluded that EPIs are associated with more lead-related complications than ENDOs, while mortality rates did not differ. However, more longitudinal multidisciplinary studies are required to validate our results. Regarding global trends to date, it seems that EPI with steroid-eluting leads is often preferred as initial PM implantation in children with smaller sizes. Endocardial leads are mostly removed via open surgery, and therefore, the endocardial pacing system can be installed as late as possible, thus preventing, to some extent, various endovascular complications in children.

### Supplementary Information

Below is the link to the electronic supplementary material.

Supplementary file1 (DOCX 415 KB)

Supplementary file2 (DOCX 57 KB)
