# Supplementary material for: Epicardial Versus Endocardial Pacing in Paediatric Patients with Atrioventricular Block or Sinus Node Dysfunction: A Systematic Review and Meta-analysis
Source: Pediatr Cardiol. 2023 Jul 22;44(8):1641–8. doi: 10.1007/s00246-023-03213-x (PMC10520152; doi:10.1007/s00246-023-03213-x)

## **Figure S1.** **PRISMA 2020 flow diagram.**

**Identification of studies via databases and registers**

Records removed *before screening*:

Duplicate records removed **(n = 1250)**

Records marked as ineligible by automation tools **(n =1328)**

Records removed due to filters that applied **(n = 588)**

Records identified from:

Databases **(n = 3693)**

PubMed, CENTRAL, Scopus, Web of Science, OpenGrey, ClinicalTrials.gov, reference lists

**Identification**

Records screened

**(n = 527)**

Records excluded

**(n = 444)**

Reports sought for retrieval

**(n = 83)**

Reports not retrieved

**(n = 21)**

**Screening**

Reports excluded:

Reason 1: Wrong population **(n = 24)**

Reason 2: No outcomes reported for both groups **(n = 8)**

Reason 3: Reviews, case reports, guidelines **(n = 12)**

Reports assessed for eligibility

**(n = 62)**

Studies included in review

**(n =18)**

Studies included in meta-analysis

**(n =15)**

**Included**

Figure S2. Risk of bias evaluation via robvis.


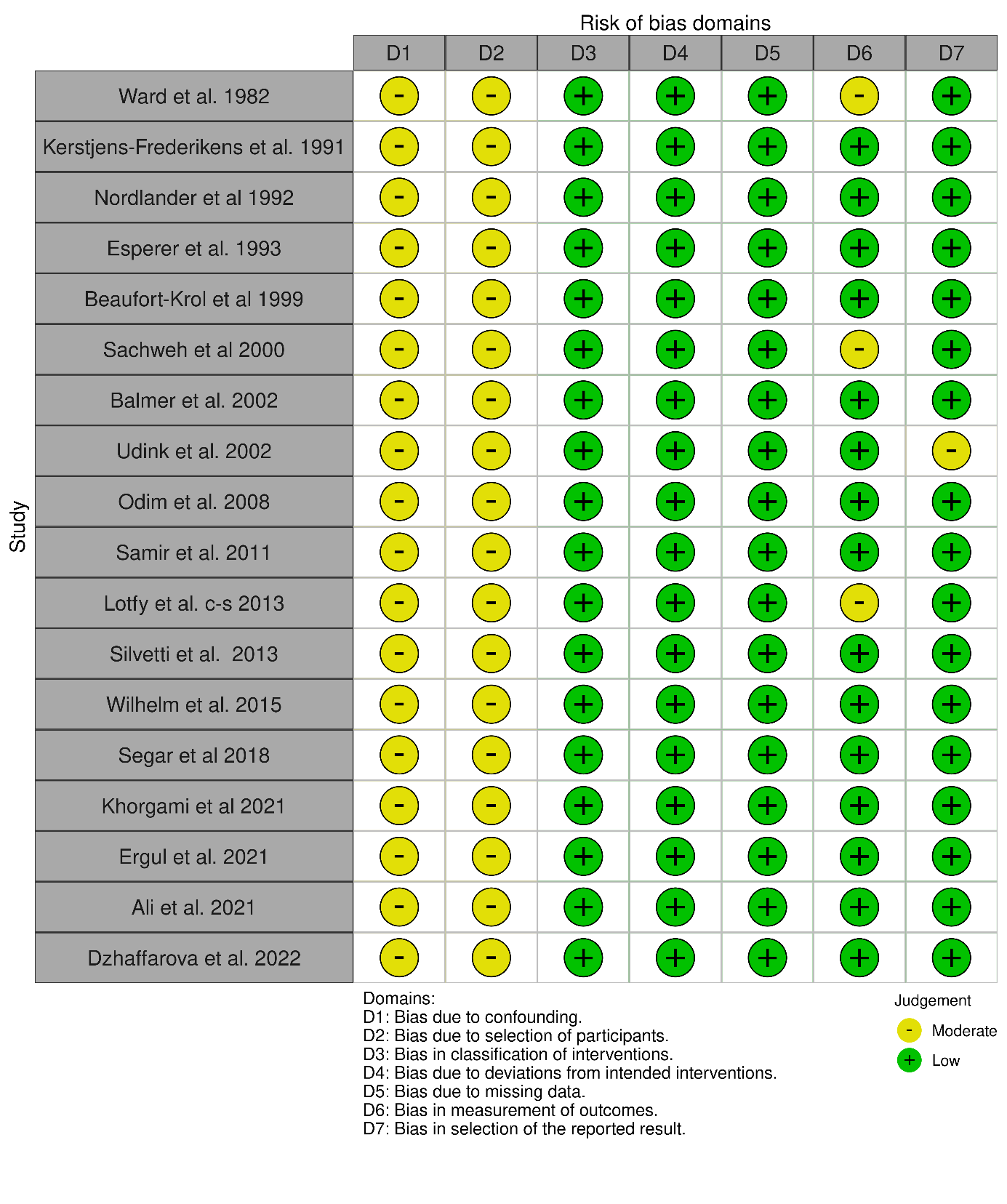


Figure S3. Primary outcome: pooled Odds ratio for PM lead failure between EPI and ENDO.


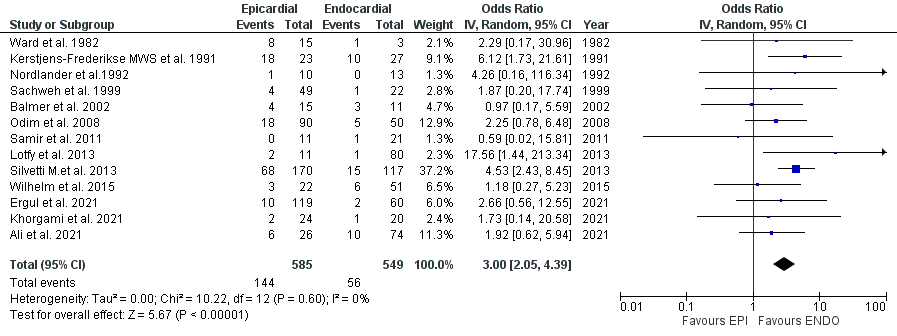


**Figure S4. Primary outcome: pooled Odds ratio for threshold rise between EPI and ENDO.**


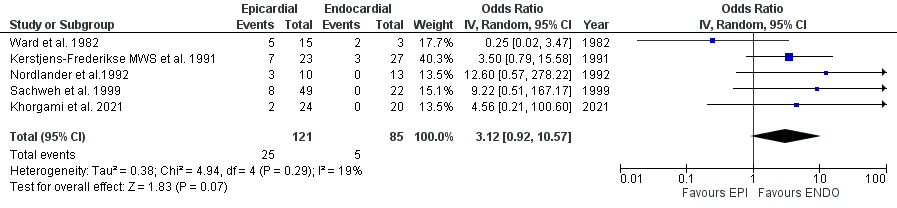


Figure S5. Primary outcome: pooled Odds ratio for post-implantation infection between EPI and ENDO.


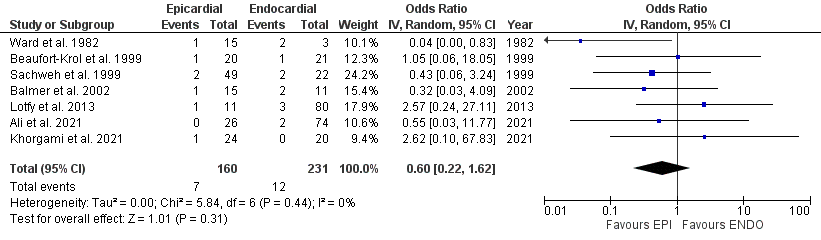


Figure S6. Primary outcome: pooled Odds ratio for battery depletion between EPI and ENDO.


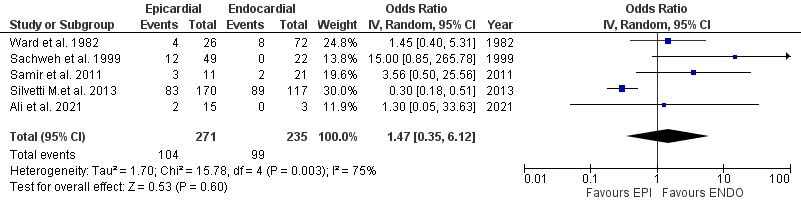


Figure S7. Secondary outcome: pooled Odds ratio for mortality between EPI and ENDO.


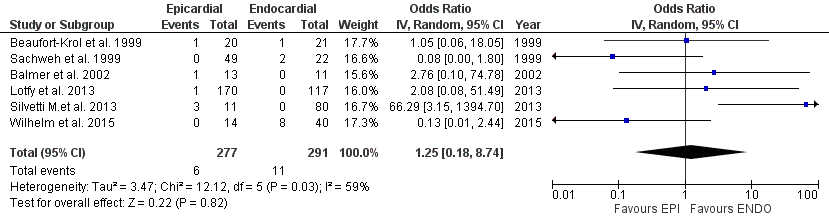


Figure S8. Sensitivity analysis for battery depletion.


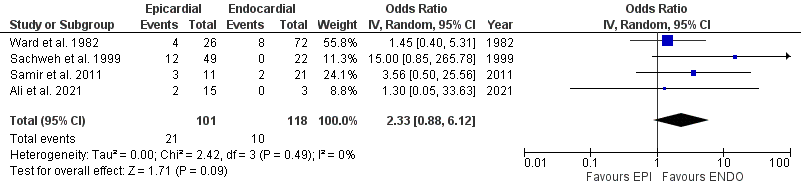


Figure S9. Sensitivity analysis for mortality.


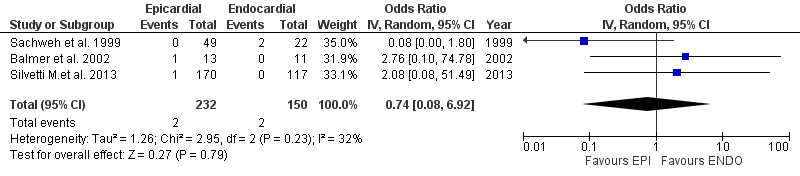

Supplement: Supplementary file 1 — Supplementary file1 (DOCX 415 KB) [file 246_2023_3213_MOESM1_ESM.docx]
