# Supplementary material for: Epicardial Versus Endocardial Pacing in Paediatric Patients with Atrioventricular Block or Sinus Node Dysfunction: A Systematic Review and Meta-analysis
Source: Pediatr Cardiol. 2023 Jul 22;44(8):1641–8. doi: 10.1007/s00246-023-03213-x (PMC10520152; doi:10.1007/s00246-023-03213-x)
Supplement: Supplementary file 2 — Supplementary file2 (DOCX 57 KB) [file 246_2023_3213_MOESM2_ESM.docx]

**Table S1. Included studies’ identifying characteristics.**

| **No** | **Study** | **Type** | **Year** | **Country** | **Population** | **Follow-up** | **EPI(%)** | **ENDO(%)** |
| --- | --- | --- | --- | --- | --- | --- | --- | --- |
| 1 | Ward et al. | RC | 1982 | London | 20 | 5.9y | 17 (85) | 3 (15) |
| 2 | Kerstjens-Frederikens et al. | RC | 1991 | Netherlands | 50 | 5.3±3.7y (6W, 3M, 6M) | 23 (46) | 27 (54) |
| 3 | Nordlander et al. | RC | 1991 | Sweden | 23 | 3.8y (0.5-6) | 10 (43.4) | 13 (56.6) |
| 4 | Esperer et al. | RC | 1993 | Germany | 33 | 4.3 ± 4.3y | 16 (48) | 17 (52) |
| 5 | Sachweh et al. | RC | 2000 | Germany | 71 | 3.4±3.8y | 49 (69) | 22 (31) |
| 6 | Beaufort-Krol et al. | RC | 2000 | Netherlands | 41 | NA | 20 (48.7) | 21 (51.3) |
| 7 | Balmer et al. | RC | 2002 | Switzerland | 32 | 10.2(0.3-20)y | 15 (46.8) | 11 (34.3) |
| 8 | Udink et al. | RC | 2002 | Netherlands | 95 | 2.4 (±2.2)y | 24 (25.2) | 71 (74.8) |
| 9 | Odim et al. | RC | 2008 | USA | 148 | 2.7± 2.4(ENDO), 2.4±1.9(EPI) | 90 (60.8) | 58 (39.2) |
| 10 | Samir et al. | RC | 2011 | Egypt | 32 | 0.25–14 (median: 2.5 y) | 11 (34.3) | 21 (65.7) |
| 11 | Lotfy et al. | CS | 2013 | Egypt | 91 | 7.16y | 11 (12) | 80 (88) |
| 12 | Silvetti et al. | RC | 2013 | Italy | 287 | 7±5y | 170 (59.2) | 117 (40.8) |
| 13 | Wilhelm et al. | RC | 2015 | Germany | 73 | 7.9y | 22 (30.1) | 51 (69.9) |
| 14 | Segar et al. | RC | 2018 | USA | 31 | NA | 23 (74.1) | 8 (25.9) |
| 15 | Khorgami et al. | RC | 2021 | Iran | 44 | NA | 24 (54.5) | 20 (45.5) |
| 16 | Ergul et al. | RC | 2021 | Turkey | 167 | 3.5y(0.5-10 EPI), 2.7y(0.5-10 ENDO) | 109 (65.2) | 58 (34.8) |
| 17 | Ali et al. | CS | 2021 | Egypt | 100 | 5.5(3-8)y | 26 (26) | 74 (74) |
| 18 | Dzhaffarova et al. | RC | 2022 | Russia | 145 | 10.2(1999-2020)y | 71 (48.9) | 74 (51.1) |

*RC: Retrospective cohort, CS: Cross-sectional

**Table S2. Population baseline characteristics.**

| **Study** | **Gender** | **Implantantion mean weight(kg)** | | **Implantation mean age(years)** | | **CAVB** | **Acquired AVB** | **SND** |
| --- | --- | --- | --- | --- | --- | --- | --- | --- |
|  | Male (%) | EPI | ENDO | EPI | ENDO |  |  |  |
| Ward et al. 1982 | NA | NA | NA | 4(1-13) | 11(10-13) | 5 | 15 | 0 |
| Kerstjens-Frederikens et al. 1991 | NA | NA | NA | 8.6 ± 4.8 | | 5 | 18 | 27 |
| Nordlander et al. 1991 | 11 (47.8) | NA | NA | 4.8 (2 days-14 yrs) | | 5 | 13 | 5 |
| Esperer et al. 1993 | 18 (54.5) | NA | NA | 5 ± 4 | 12 ± 5 | 2 | 24 | 6 |
| Sachweh et al. 1999 | 39 (54.9) | 15.0 ±11.2 | 23.9 ±11.4 | 4.5± 4.2 | 7.0 ± 3.6 | 22 | 49 | 0 |
| Beaufort-Krol et al. 2000 | 20 (48.7) | NA | NA | 7.6 ± 6.5 | 11.7 ± 4.7 | 23 | 9 | 0 |
| Balmer et al. 2002 | 17 (53.1) | NA | NA | 0.4(0–16·5) | 7.3(0–16·5) | 5 | 15 | 0 |
| Udink et al. 2002 | 48 (50.5) | 17.75 | 26.05 | 5.6 | 8.2 | 40 | 41 | 10 |
| Odim et al. 2008 | NA | 19.7± 17.6 | 38.1± 18.1 | 5.5±5.3 | 11.0± 4.3 | 41 | 71 | 36 |
| Samir et al. 2011 | 18 (56.2) | 21.6 ± 13.8 | | 5.7 ± 3.8 | | 13 |  | 3 |
| Lotfy et al. 2013 | 59 (64.8) | 12 | | 2.3 (0.09-12) | | 31 | 54 | 6 |
| Silvetti et al. 2013 | 166 (57.8) | NA | NA | 4 (1 – 9) | 8 (3 – 14) | 172 | 0 | 115 |
| Wilhelm et al. 2015 | 46 (63.0) | 10.6 | 26.5 | 2.2 | 8.3 | 32 | 41 | 0 |
| Segar et al. 2018 | 15 (48.3) | 42.2± 27.5 | 40.0± 21.9 | 13.8 ± 11 | 11.4± 9.4 | 5 | 0 | 26 |
| Khorgami et al. 2021 | 18 (40.9) | NA | NA | 2 (0.1–16 | 5 (1.2–15 | 11 | 32 | 1 |
| Ergul et al. 2021 | 96 (57.4) | 14(2.7-85) | 45 (21-76) | 2.41 (0.3-18) | 12.3 (5.3-18) | 71 | 95 | 13 |
| Ali et al. 2021 | 51 (51.0) | 20.85 ± 4.81 | 56.77± 19.00 | 6.10 ± 2.23 | 14.85 ± 3.97 | 52 | 46 | 2 |
| Dzhaffarova et al. 2022 | NA | NA | NA | ΝΑ | ΝΑ | 103 | 0 | 25 |

*±SD, NA: Not available

Table S3. GRADE rating of outcomes.

| **Outcomes** | **No of studies** | **Risk of bias** | **Inconstistency** | **Indirectness** | **Imprecision** | **Publication bias** | **Overall certainty of evidence** |
| --- | --- | --- | --- | --- | --- | --- | --- |
| PM-lead failure (imp.) | 11 OC  2 CS | Not serious | Not serious | Not serious | Not serious | None | Low |
| Threshold rise (imp.) | 5 OC | Not serious | Not serious | Not serious | Not serious | NA | Low |
| Infection (crit.) | 5 OC  2 CS | Not serious | Serious | Not serious | Not serious | NA | Low |
| Battery depletion (imp.) | 4 OC  1 CS | Not serious | Serious | Not serious | Not serious | NA | Low |
| Mortality (crit.) | 5 OC  1 CS | Not serious | Serious | Not serious | Serious | NA | Very Low |
| Hemothorax (crit.) | 2 OC | Not serious | Not serious | Not serious | Very serious | NA | Very Low |
| Venous occlusion (crit.) | 1 OC | Not serious | Not serious | Not serious | Serious | NA | Low |

*OC: Observational cohort study, CS: Cross-sectional, ΝΑ: Not available
